# Leaching of Titanium Dioxide Nanomaterials from Agricultural Soil Amended with Sewage Sludge Incineration Ash: Comparison of a Pilot Scale Simulation with Standard Laboratory Column Elution Experiments

**DOI:** 10.3390/ma15051853

**Published:** 2022-03-01

**Authors:** Boris Meisterjahn, Nicola Schröder, Jürgen Oischinger, Dieter Hennecke, Karlheinz Weinfurtner, Kerstin Hund-Rinke

**Affiliations:** 1 Fraunhofer Institute for Molecular Biology and Applied Ecology IME, Auf dem Aberg 1, 57392 Schmallenberg, Germany; boris.meisterjahn@ime.fraunhofer.de (B.M.); nicola.schroeder@ime.fraunhofer.de (N.S.); karlheinz.weinfurtner@ime.fraunhofer.de (K.W.); kerstin.hund-rinke@ime.fraunhofer.de (K.H.-R.); 2 Fraunhofer Institute for Environmental, Safety and Energy Technology UMSICHT, An der Maxhütte 1, 92237 Sulzbach-Rosenberg, Germany; juergen.oischinger@umsicht.fraunhofer.de

**Keywords:** nano titanium dioxide (nTiO_2_), engineered nanomaterial (ENM), sewage sludge incineration (SSI), ENM containing sewage sludge ash (SSA), leaching, column elution, agricultural use

## Abstract

Nanoscale titanium dioxide (nTiO_2_ (Hombikat UV 100 WP)) was applied to sewage sludge that was incinerated in a large-scale waste treatment plant. The incineration ash produced was applied to soil as fertilizer at a realistic rate of 5% and investigated in pilot plant simulations regarding its leaching behavior for nTiO_2_. In parallel, the applied soil material was subject to standard column leaching (DIN 19528) in order to test the suitability of the standard to predict the leaching of nanoscale contaminants from treated soil material. Relative to the reference material (similar composition but without nTiO_2_ application before incineration) the test material had a total TiO_2_ concentration, increased by a factor of two or 3.8 g/kg, respectively. In contrast, the TiO_2_ concentration in the respective leachates of the simulation experiment differed by a factor of around 25 (maximum 91.24 mg), indicating that the added nTiO_2_ might be significantly mobilisable. Nanoparticle specific analysis of the leachates (spICP-MS) confirmed this finding. In the standard column elution experiment the released amount of TiO_2_ in the percolates between test and reference material differed by a factor of 4 to 6. This was also confirmed for the nTiO_2_ concentrations in the percolates. Results demonstrate that the standard column leaching, developed and validated for leaching prediction of dissolved contaminants, might be also capable to indicate increased mobility of nTiO_2_ in soil materials. However, experiments with further soils are needed to verify those findings.

## 1. Introduction

Engineered nanomaterials (ENMs) applied, e.g., in consumer products, can be released to the environment during their use (e.g., release of silver nanoparticles (AgNPs) from facade painting [1] or TiO_2_-nanoparticles (TiO_2_-NPs) from sunscreens [2]), while, after use, a major fraction of the ENMs is supposed to be released to wastewater streams [3] and, furthermore, becomes attached to sewage sludge during wastewater treatment [4,5]. The majority of sewage sludge is incinerated and ends up in landfills [6].

Sewage sludge incineration ash (SSA) can be used for the production of phosphorous fertilizer (e.g., according German fertilizer ordinance [7]). This recycling route will be increased in the future because the recovery of phosphorus from sewage sludge and sewage sludge ash is required by law in Germany and other countries of the EU. 

The use of materials in soils always requires a specific assessment to prevent soil contamination by re-use of waste materials. Integral part of the assessment is the determination of the source strength from those materials for leaching of contaminants. This is performed, e.g., by a standard column elution according to DIN 19528 for dissolved heavy metals and a set of organic substances.

So far, very little has been published regarding the release of nanoparticles from SSA. In a very recent study, Wielinski et al. [8] investigated the release of different nanomaterials from SSA in column experiments, but the column experiments described therein are very different to the standard column elution used for evaluation of source strength in the scope of regulation. Further, laboratory column experiments need references from the “real world” to assess the robustness of the laboratory results. However, we could not find any study regarding the release of nanomaterials from SSA after use in agricultural soils as fertilizer. Respective studies mostly focus on heavy metal contaminations (not in the form of NPs) or availability of phosphorous from SSA. Thus, there were two knowledge gaps identified for our study: (i) the question of leaching of NPs that might enter agricultural soils by fertilization with NP-contaminated SSA under realistic and environmentally relevant conditions and (ii) the predictive power of a column elution procedure actually used in the German soil protection ordinance [9] for NP leaching. 

These gaps were addressed by the present study. In order to investigate the potential leaching of a representative ENM nano titanium dioxide (nTiO_2_) from SSA applied to soils, combined elution experiments in pilot and laboratory scale were performed. nTiO_2_ was selected as test material, as it is among the most produced nanomaterials [10,11] and was used, e.g., in suncreens [2], as photocatalyst [12] or food additives [13]. Ash material was produced in a large-scale waste incinerator from incineration of sewage sludge amended with nTiO_2_. The resulting SSA was mixed with a reference soil at a rate of about 5%. This is about a factor of 10 greater than the maximum allowed application rate [14] and was considered to be a worst case scenario. The SSA amended soil was used for a pilot scale bioreactor trial in order to investigate nTiO_2_ leaching from the material under controlled realistic conditions. In a timelapse experiment, three annual summer/winter cycles were simulated within 250 days to verify the influence of seasons on the leaching behavior. Those pilot scale simulations were accompanied by standard laboratory column elution experiments. Based on the results obtained in both tests, the suitability of the standard column elution with regard to a prediction of nTiO_2_ leaching from soil treated with SSA should be determined. In case of a significant correlation, the standard laboratory column elution could be extended for risk assessment of nanomaterial leaching from SSA amended soils. So far, this has not been considered in soil protection, since transport of nanomaterials in soil cannot be described by common leaching models, as the sorption theory used for dissolved chemicals does not apply for nanomaterials [15].

## 2. Materials and Methods 

### 2.1. Preparation of ENM Containing Sewage Sludge Incineration Ash (SSA) 

For the production of the ENM-containing sewage sludge ash (SSA), the product Hombikat UV 100 WP from Co. VENATOR was used. The aqueous Hombikat UV 100 WP dispersion consists of 42% (*w*/*w*) nTiO_2_ and about 6.5% (*w*/*w*) of poly acrylate acting as stabilizer. As a basis for the formulation of Hombikat UV 100 WP, nTiO_2_, in the form of the product Hombikat UV 100 with a primary particles size of <10 nm, was employed. Further information can be found in Börner et al. [16] and Oischinger et al. [17]. The application of Hombikat UV 100 WP lies mainly in the photocatalytic area, as the nTiO_2_ is present in the anatase modification.

The sewage sludge incineration was performed at the Sewage Sludge Incineration Plant (SSIP) of the waste water treatment plant ZVK Steinhäule at Neu-Ulm. Annually a wastewater amount of 440,000 population equivalents is cleaned in the plant, resulting in 10,000 t of sewage sludge (dry matter (DM)) and 2500 t SSA [18]. The SSIP consists of a centrifuge, a dryer, a fluidised-bed incinerator with selective non-catalytic NO_x_ reduction, an electrostatic precipitator, a 2-stage scrubber and an activated-carbon reactor with fabric filter. A schematic diagram of the plant is depicted in [16]. 

For the production of the reference ash material on the 1st day, the average amount of sewage sludge mounted up to 2171 kg h^−1^ (DM) and, for the day with nTiO_2_ injection, on the 2nd day, up to 2120 kg h^−1^ (DM). During the measurement with nTiO_2_ injection, 630 kg of nTiO_2_ dispersion was added to the sewage sludge with a peristaltic pump over 6 h. On both days, the measured background concentration of titanium was <0.1 wt%. Hence, an augmentation of titanium in the sewage sludge of about 1.25 wt% was achieved by the injection of the dispersion. The SSA was sampled on the basis of the recommendations of LAGA PN98 [19] for moving waste, as far as they were applicable to the large scale plant [20]. 

### 2.2. Simulation Experiments

#### 2.2.1. Leaching in Pilot Scale Simulation Reactors

For investigation of the release of nanoparticles from SSA applied in soil-related applications, experiments in pilot-scale simulation reactors were conducted. For this purpose, the incineration residue (with and without treatment with nTiO_2_) was mixed with an agricultural soil (refesol 04-A, details of the soil characteristics see Table 1). 

**Figure 1:**
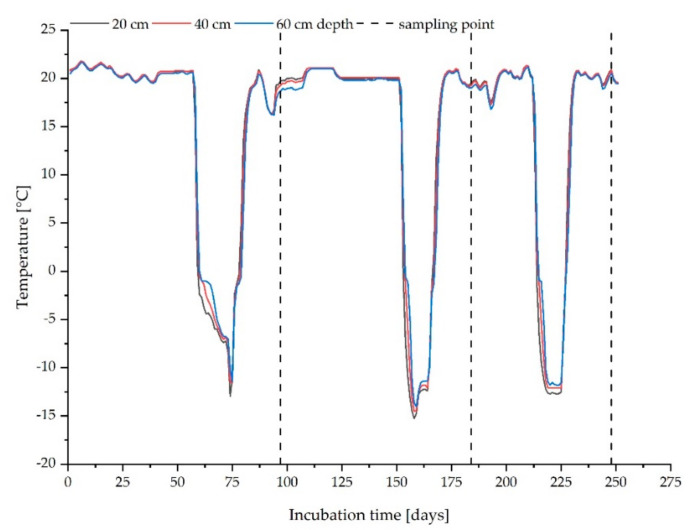
Temperature curve over the entire test period with three annual cycles, measured at 20, 40 and 60 cm distance from the edge of the reactor with treated soil ash/mixture. Dotted line marks the three sampling points after the winter phases (23 January, 24 April and 27 June).

The sandy soil with higher organic carbon content was selected in order to simulate worst case conditions regarding potential nanoparticle release and mobility [21,22]. Per reactor, 700 kg of soil material (dry weight basis) were mixed with an amount of SSA, representing 5% with regard to the dry weight of the soil material, corresponding to approximately 190 t sludge ash per ha, taking into account an assumed plough share depth of 25 cm. At mixing, the soil was adjusted to a water content representing 50% of its water holding capacity (WHC), being optimal for microbial activity. The mixture was then subjected to an experiment simulating at least three annual seasonal cycles during a total incubation time of 250 days. Thus, the soil/ash mixture was incubated at 20–22 °C simulating a summer phase, followed by freezing of the mixture to −10 to −15 °C final temperature. The soil in the reactor was kept at this temperature for 14 days (simulated winter period). After each winter period and thawing of the soil/ash mixture, the soil was dug up and samples were taken for the column elution experiments (seasonal cycles and sampling dates see Figure 1). The soil was irrigated over a period of 12 days with 10 L portions until approximately 5 L of seepage/leachate water was collected. The leachate was analysed for total titanium concentration by ICP-OES after microwave assisted digestion and, in addition, was subjected to nanoparticle specific analysis by single particle (sp)ICP-MS. For the next cycle, the soil was again dried to a water content of 50% WHC by digging the soil up several times and leaving the reactor surface open while incubating at 20 ± 2 °C. After reaching the desired water content, the reactor was closed again and the next cycle started. 

#### 2.2.2. Column Elution Tests

At three time points, samples of the soil/ash mixture in the pilot-scale reactors were sampled and filled into glass columns (diameter 5.5 cm) according to the instructions given by the standard DIN 19528 [23] for soil column leaching. At both ends of the glass columns, quartz sand was used as a filtration layer. The column was then subjected to an elution according to DIN 19528 [23]. The column was saturated with deionized water from the bottom to the top layer. After saturation, the column was eluted with deionized water and eluate samples taken at five different solid/water ratios (0.3, 1, 2, 4 and 10 L/kg). The titanium concentration in the eluate was determined as described in Section 2.3.1 without any previous eluate filtration or centrifugation. The amount of soil per soil column and the percolation rates for saturation and percolation are presented in Table 2. The percolation rates were calculated according to DIN 19528:

(1) $$q=\frac{l\;\times \;\pi \;\times \;r^{2}\;\times \;n}{t\;\times \;60}$$

with *q* = percolation rate (mL min^−1^); *l* = length of soil column; *r* = inner radius of column; *n* = porosity (0.43); *t* = time (2 h for saturation, 5 h for percolation).

### 2.3. Chemical Analysis

#### 2.3.1. TiO_2_ Concentrations in Reactor Leachates and Column Eluates

For determination of the total concentration of titanium in the leachates of the reactors, as well as for the column eluates, 10 mL of the respective sample was filled into a Teflon^TM^ digestion vessel (MLS, Leutkirch, Germany) and evaporated at 105 °C to dryness. The residue was then mixed with 4.8 mL of HNO_3_ (69%, supra pure, Roth) and 0.2 mL hydrofluoric acid (HF, 40%, supra pure, Roth) and digested in a microwave (Ultraclave II, MLS, Leutkirch, Germany; digestion parameters: 220 °C, 30 min, 100 bar). After digestion, 1 mL boric acid was added in order to complex the remaining HF; the mixture filled up to a final volume of 15 mL with ultrapure water. The concentration of Ti in the solution was then determined by ICP-OES (wavelength 334.941 nm, Instrument: Agilent 5110, Agilent technologies). The obtained results were converted into TiO_2_ concentrations under the assumption that no other titanium-containing phase was present in the samples. 

#### 2.3.2. TiO_2_ Concentrations in Soil/Ash-Mixed Samples

For determination of total titanium concentrations in mixtures of soil and sewage, sludge ash was applied for simulation in the pilot-scale reactors; in addition, column elution experiments, 5 aliquots of 5 g (fresh weight) each, were taken and mixed to obtain a representative sample. The samples were dried at 105 °C until constant weight. Aliquots of approximately 200 mg of the dried residues were then weighted in Teflon^TM^ digestion vessels and 1 mL HF (40% supra pure, Roth) and 4 mL HNO_3_ (69%, supra pure, Roth) added. The samples were subjected to microwave-assisted digestion (Ultraclave II, MLS, digestion parameters: 220 °C, 30 min, 100 bar). After digestion, 5 mL of boric acid was added to complex remaining HF and the digestate filled up to a final volume of 15 mL with ultrapure water. The solution was analysed for titanium by ICP-OES at a wavelength of 334.941 nm. 

#### 2.3.3. Single Particle (sp)ICP-MS Analysis of Reactor Leachates and Column Eluates

In addition to the determination of the total titanium concentration, number-based particle size distributions were determined by single particle inductively coupled plasma mass spectrometry (spICP-MS) [24,25]. The aqueous samples were directly measured without any further sample preparation, except for dilution with ultrapure water. The analyses were performed using a triple-quadrupole ICP-MS instrument (ICP-QQQ-MS, Agilent 8900, Agilent Technologies, Waldbronn, Germany). The dwell time in the single particle measurement mode of the ICP-MS was set to 100 µs and time-resolved signals were recorded on the selected *m*/*z* for 60 s. Peak detection and integration was conducted automatically by the Agilent MassHuntersoftware. Conversion of signal heights of particle spikes into particle sizes were performed by application of a calibration with a dissolved Ti standard. To apply a dissolved calibration for size calculation of nanoparticles from their signal spikes, the nebulization or transport efficiency in the interface was determined by analyzing a gold nanoparticle standard of known concentration and size [24]. Dispersions of 60 nm gold nanoparticles (AuNPs 60 nm, BBI solutions, Kent, UK) were used for the determination of the nebulization efficiency and were prepared freshly on the day of measurement. The samples were diluted in ultrapure water by a factor of 10^2^–10^5^ for measurement in order to reach a particle concentration of 200–2000 particle events per minute. 

Due to possible interferences for the most abundant titanium isotope ^48^Ti caused by the calcium isotope ^48^Ca present in the leachate and eluate matrices, titanium was measured in the MS/MS mode with ammonia as reaction gas (10% NH_3_ + 1 mL/min He). Thus, titanium was measured as [^48^TiNH]^+^ with a *m*/*z* of 63. The threshold between background and particle signals was defined based on visual inspection of the measured signal distributions. The conversion of signal distributions into number-based size distributions, as well as particle number concentrations, was performed by the Agilent MassHunter software. 

## 3. Results

### 3.1. Characterisation of the Produced SSA

The two SSA produced differed in their titanium content as expected. For the SSA, from the reference day, an average titanium concentration of about 0.3 wt% (dry mass (DM)) with a standard deviation (SD) of 0.04 was determined, whereas the SSA treated with nTiO_2_ had a mean value of 2.9 wt% (DM) with an SD of 0.19 [26]. Hence, the titanium content in the nTiO_2_-SSA was increased compared to the reference measurement by a factor of about 9.7 [26]. Further information on characterisation of the used SSA can be found elsewhere [26]. 

### 3.2. Leaching from Pilot Scale Simulation Reactors

#### 3.2.1. Determination of Total Titanium Content in Mixtures of Soil/SSA and in Leachates 

**Figure 2:**
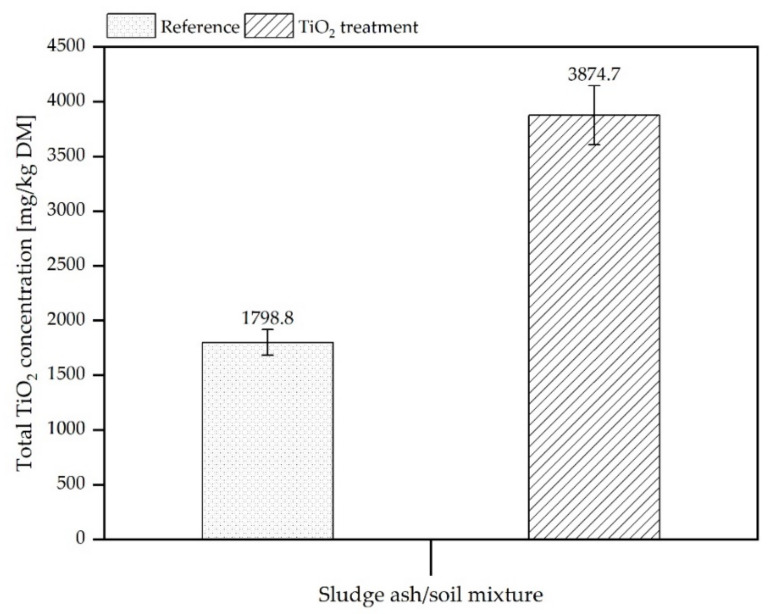
Total TiO_2_ concentrations (converted Ti-concentrations) in sludge ash/soil mixtures. left column: Concentration in samples using sludge ash without treatment with nTiO_2_, right column: Concentration in samples using SSA treated with nTiO_2_. Error bars refer to standard deviation of four subsamples that were digested and analysed.

Figure 2 shows the determined total titanium concentrations in both mixtures of soil with SSA with additional nTiO_2_ or without nTiO_2_ treatment (reference samples) which were filled into the pilot scale reactors. The SSA/soil mixture containing nTiO_2_-treated ash shows a titanium concentration elevated by a factor of approximately two compared to the reference sample. Thus, at least this ratio should also be found in the leachates. 

**Figure 3:**
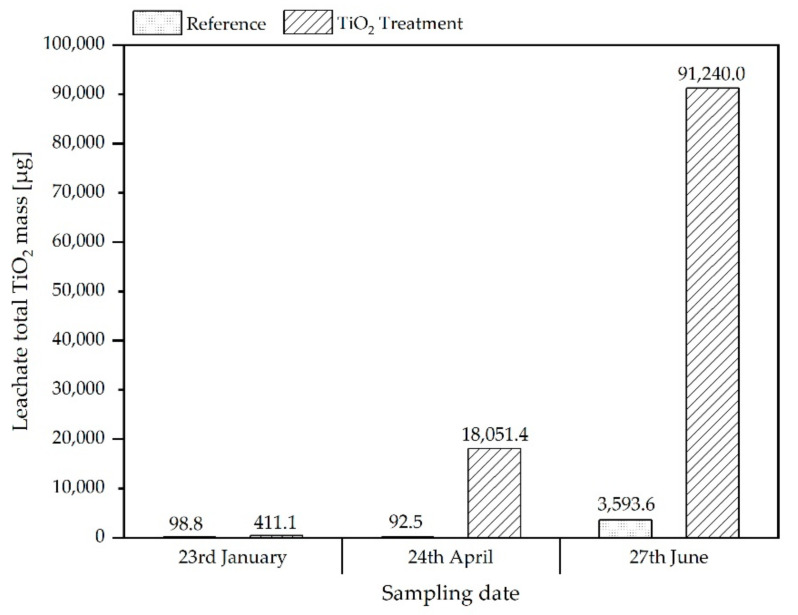
Total TiO_2_ content (derived from Ti-concentrations) determined in leachates from pilot scale simulation reactors collected after watering of the soil at end of the respective preceding winter phase.

However, as shown in Figure 3, the titanium/converted TiO_2_ content in the leachates of treatment and reference reactors collected at the end of the three simulated annual seasonal cycles was found to be higher for the treated samples compared to the references by a factor of ~25. Titanium-containing particles are released from the treatment reactor in a significantly higher amount than expected from the total titanium content of the respective mixtures, thus, indicating that the added nTiO_2_ (or titanium containing particles) might be more mobilisable than the titanium-bearing particles (most likely also being TiO_2_) already present in the soil and sludge ash.

#### 3.2.2. SpICP-MS/MS Analyses of Leachates

**Figure 4:**
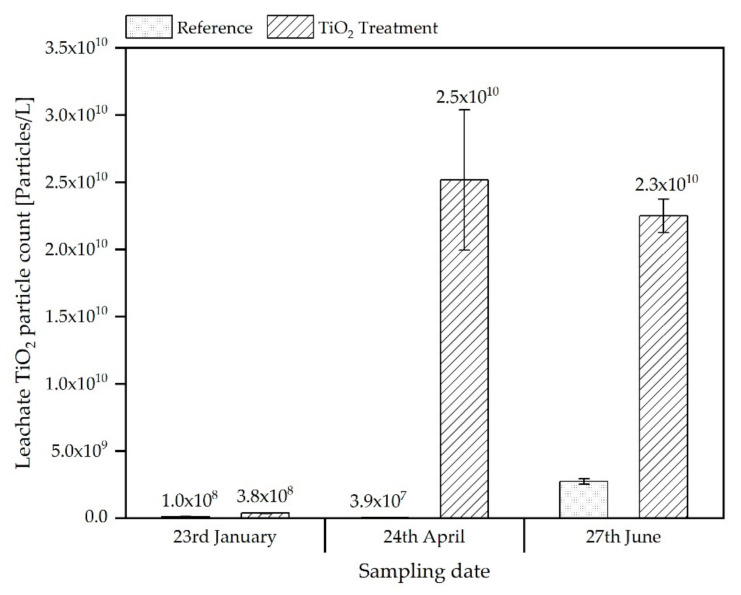
TiO_2_ particle concentrations (Particles/L) determined in leachates from pilot scale simulation reactors collected after watering of the soil at end of the respective preceding winter phase.

The significantly higher release of titanium in the leachates from the treatment reactor is also visible in the nanoparticle specific analysis of the leachates by spICP-MS, as shown in Figure 4. The higher TiO_2_ particle discharge in leachates after the first winter period (23 January) is 3.7 times higher in the treated reactor and rises to 640 times after the second simulated winter phase (17 April) and drops to 8.2 times after the third winter phase (27 June) (Figure 4). Again, the differences in discharge are much higher than could be expected from the total Ti-content determined for the soil/ash mixtures applied to the two reactors. 

### 3.3. Column Elution Experiments

The percolate samples obtained from standard column eluate experiments were analysed analogously to the leachates from the pilot scale experiments for their total TiO_2_ content (ICP-OES analysis after HF digestion) and particle size distribution (spICP-MS).

**Figure 5:**
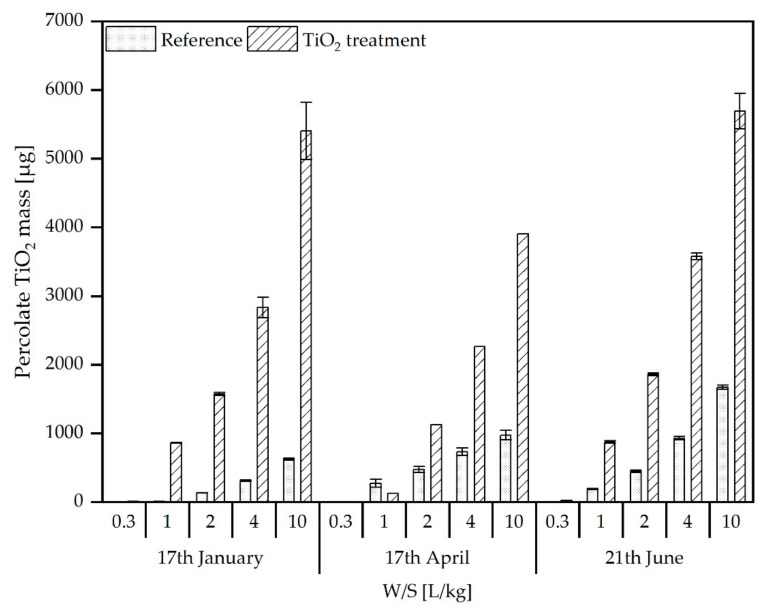
Total TiO_2_ content (µg) in column percolates from reference and nTiO_2_ treated SSA/soil mixtures out of pilot scale simulation reactors collected at three different sampling times.

Figure 5 shows the TiO_2_ content in the percolates of soil/ash-material taken at end of the three simulated annual seasonal cycles from the treatment and reference reactor to investigate the behaviour during soil-related use. In the percolates, an increased TiO_2_ load was detected for the treated SSA/soil mixture compared to the reference mixture. In contrast to the analyses of the reactor leachates, the ratio between TiO_2_ released from nTiO_2_-treated material and the reference material was found to be more or less stable during all three sampling times. Already, percolates from the first sampling (17 January) showed increased TiO_2_ contents in the nTiO_2_ treated SSA/soil mixture. This was most likely due to the very high water:solid ratios used in the column elution compared to the pilot scale experiment. However, the maximum TiO_2_ concentrations determined are in the same range as the maximum concentration found in the pilot scale experiment.

**Figure 6:**
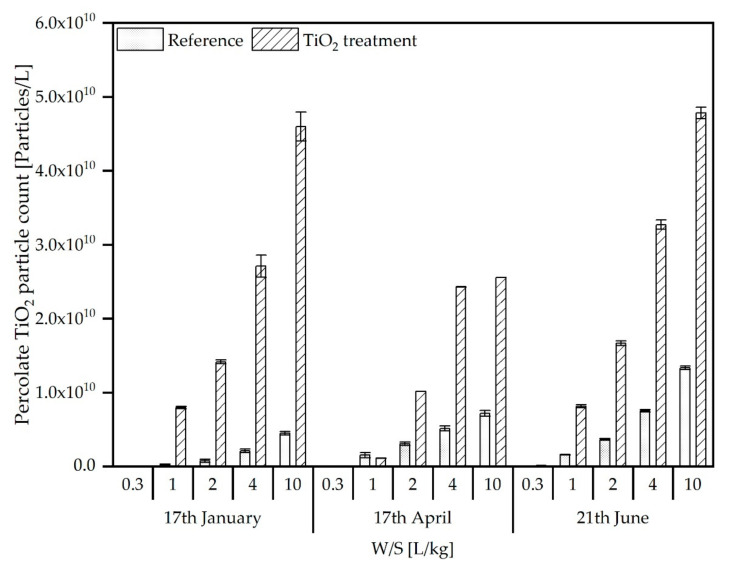
nTiO_2_ particle concentrations (Particles/L) determined in column percolates from reference and nTiO_2_ treated SSA/soil mixtures out of pilot scale simulation reactors collected at three different sampling times.

The spICP-MS/MS analyses of the column percolates (reference and nTiO_2_ treatment) show a higher nTiO_2_ particle discharge from the nTiO_2_ treated SSA/soil mixture (Figure 6). The particle number in the percolates of the reference and TiO_2_ treated SSA/soil mixtures increases in both cases with the water/solid ratio (W/S). In the TiO_2_-treated percolates, the particle concentration at a W/S of 10 L/kg at the first sampling (17 January) extend concentration in the reference percolates by a factor 10. During the following samplings (17 April and 21 June), particle counts in the nTiO_2_ percolates extend the reference percolates by a factor of 4. In general, the nTiO_2_ analysis confirms the findings of the total TiO_2_ analysis, indicating that most of the TiO_2_ determined was due to leached nTiO_2_.

## 4. Discussion

In this study, nTiO_2_ was used as an exemplary ENM for the preparation of ENM-containing SSA produced for the soil simulation experiments. In the simulation of agricultural use of this nTiO_2_ amended SSA, a clearly increased release of nTiO_2_ was found in the leachates of the pilot scale simulation reactors after a certain time compared to a reference ash from non-amended sewage sludge. The amount of release was significantly higher than could be expected due to the differences in the total TiO_2_ contents in the soil-ash mixture relative to the reference ash. A cause for the comparatively high releases could not be determined within the project. After three freezing and thawing cycles, the amount of TiO_2_ released from the reference was increased significantly, indicating mobilization by changes of the soil structure due to frost wedging. This is also observed in increasing amounts of TiO_2_ in the column experiments throughout the three samplings. A difference between the leaching from the simulation reactors and standard column elution is the significantly higher ratio between TiO_2_ released from reference and treated SSA in the reactors compared to the ratios observed for the column experiments, which were nearer to the ratio of total Ti contents of both materials (Figure 2) at the end of the study. For instance, while, for the reactors, the release of TiO_2,_ expressed in terms of total concentrations, from treated samples compared to the reference, was elevated by a factor of approx. 25, the analogous ratio for the standard column experiments was found to be approx. 3 (Figure 3 and Figure 5). The differences are not that pronounced if the particle counts from spICP-MS analyses are assessed with factors of approx. 10 (leachates of treated-against-reference reactors) and 4 (column percolates of treated-against-reference soil/ash-mixtures). Thus, the particle counts agree with the total titanium on a relative scale for the column experiments, whereas, there seems to be an underestimation for the reactor leachates. This might be explained by losses of, e.g., bigger particles, which would significantly contribute to the total titanium mass in the sample, due to sedimentation prior to measurement or the underestimation of the number of bigger particles due to the applied analysis time of 60 s. In contrast, the total Ti determination after digestion captures the complete amount of Ti in the sample. However, in principle, the spICP-MS analyses give similar results to the determination of the total concentrations and can possibly be a fast alternative analytical method that, additionally, provides also information on particle sizes. 

Looking further and in more detail to the obtained data, some differences can be noticed not only between the elution behavior of the test materials but also between the elution procedures itself. The elution procedure in the column and the simulation experiment were very different. In the column elution, within 7 days, a water to solid ratio of 10 L per kg soil material was applied. In the simulation, the maximum water to solid ratio used was 0.5 L per kg of soil material over a time of 250 days. This results in very different flow velocities in both approaches; it is not surprising that these huge differences in the elution conditions give different results in eluate analysis.

This significant influence of different elution conditions is strikingly visible if the released TiO_2_ amounts are related to the total amount of soil/ash mixture in the respective experiments. For instance, for the last sampling date, after three seasonal cycles, around 130 µg/kg (released amount (Figure 3) referred to 700 kg of soil/ash (dry weight) material per reactor) was released from the reactor, while, for the column elution experiments, 5172 µg/kg (TiO_2_ content in percolate referred to 1.1 kg soil/ash per column) was released. Thus, the column leaching experiments are overestimating the leaching for this kind of particle in comparison to the more realistic simulation experiments. 

The higher relative release of TiO_2_ from reference samples in the column experiments indicates that the natural “background” TiO_2_ might be attached differently to the soil matrix, as it is more easily mobilized under the experimental conditions compared to the simulation experiments. 

Therefore, the main finding of the study is that the standard column experiment according to DIN 19528 might be suitable to show increased mobility and emissions of nTiO_2_ during soil-related recycling. This finding is confirmed by simulation experiments under more realistic conditions. It proves the general suitability of the standardized column elution method for predicting an increased release of nTiO_2_ from a soil-ash mixture. 

However, due to the limitations of a pilot scale experiment, which does not allow to test a reasonable number of different soils that would be needed for a more general statement, results cannot be generalized at this point. However, they indicate that increased leaching of ENM measured in a standard soil column approach relative to a reference material might serve as reasonable prediction of increased leaching of ENM under realistic environmental conditions. As a next step, a larger data set should be determined on both SSAs using a set of different soils for standard column elution to obtain the influence of different soil matrices. 

## 5. Conclusions

The results of this study demonstrate that a standard soil column experiment might be suitable to reliably indicate an increased mobility of nTiO_2_ from SSA mixed with agricultural soil relative to a respective reference material containing only “background” TiO_2_. However, due to the limited data set of so far just one soil general conclusions cannot be made at this point. Still, the result is important for future exposure assessment, as it might offer an experimental laboratory tool to determine the risk of leaching from a recycling material. 

The use of an accepted standard (DIN 19528) for the determination of the mobility offers the chance to get a new standard for testing ENM in soil material just by extension of the scope of an existing standard. In order to establish effective thresholds and to make them available for regulatory practice, respective standards for laboratory use are required. However, further research is necessary to verify current results and to base findings on a broader database before standardization processes might start. Laboratory elution experiments could give a useful contribution to the classification and safer use of incineration ashes, which contain not only potential pollutants but also many components that are too valuable for landfill disposal. Even if no direct transfer from the laboratory elution tests on the simulation experiments can be derived, the general concept is confirmed.

## Figures and Tables

**Table 1:** Refesol 04-A soil characteristics (loamy sand).

Soil Type	Soil Texture (DIN)			Soil Texture	C_org_ (%)	N_total_ (g/kg)	pH_CaCl_2__	CEC_eff_ (mol/kg)	WHCmax (g/kg)
	Sand (%)	Silt (%)	Clay (%)						
Refesol 04-A	79.7	14.9	5.4	loamy sand	3.04	1.76	5.11	0.0412	346

**Table 2:** Mass of soil per soil column and percolation rates for saturation and percolation.

Soil	Mass per Column	Percolation Rate (mL min^−1^)	
	(g)	Saturation	Percolation
RefeSol 04-A	1100	2.64	1.06

## Data Availability

Restrictions apply to the availability of these data. Data was obtained from the project funded by the German Environment Agency (FKZ 3712 33 327) and are available from the corresponding authors with the permission of the German Environment Agency.

## References

[1] Kaegi R., Sinnet B., Zuleeg S., Hagendorfer H., Mueller E., Vonbank R., Boller M., Burkhardt M. (2010). Release of silver nanoparticles from outdoor facades. Environ. Pollut..

[2] Gondikas A.P., von der Kammer F., Reed R.B., Wagner S., Ranville J.F., Hofmann T. (2014). Release of TiO_2_ nanoparticles from sunscreens into surface waters: A one-year survey at the old Danube recreational Lake. Environ. Sci. Technol..

[3] Keller A.A., McFerran S., Lazareva A., Suh S. (2013). Global life cycle releases of engineered nanomaterials. J. Nanopart. Res..

[4] Westerhoff P., Song G., Hristovski K., Kiser M.A. (2011). Occurrence and removal of titanium at full scale wastewater treatment plants: Implications for TiO_2_ nanomaterials. J. Environ. Monit..

[5] Kaegi R., Voegelin A., Sinnet B., Zuleeg S., Hagendorfer H., Burkhardt M., Siegrist H. (2011). Behavior of metallic silver nanoparticles in a pilot wastewater treatment plant. Environ. Sci. Technol..

[6] Wiechmann B., Dienemann C., Kabbe C., Brandt S., Vogel I., Roskosch A. (2015). Sewage Sludge Treatment in Germany; Umweltbundesamt, Dessau-Roßlau. https://www.umweltbundesamt.de/sites/default/files/medien/378/publikationen/sewage_sludge_management_in_germany.pdf.

[7] (2012). DüMV, Verordnung über das Inverkehrbringen von Düngemitteln, Bodenhilfsstoffen, Kultursubstraten und Pflanzenhilfsmitteln (Düngemittelverordnung–DüMV) (German Fertilizer Ordinance); Bundesgesetzblatt (Federal Law Gazette) Jahrgang 2012 Teil I Nr. 58. https://www.gesetze-im-internet.de/d_mv_2012/.

[8] Wielinski J., Gogos A., Voegelin A., Müller C.R., Morgenroth E., Kaegi R. (2021). Release of gold (Au), silver (Ag) and cerium dioxide (CeO_2_) nanoparticles from sewage sludge incineration ash. Environ. Sci. Nano.

[9] (1999). BBodSchV, Bundes-Bodenschutz- und Altlastenverordnung vom 12. Juli 1999 (BGBl. I S. 1554), die Zuletzt durch Artikel 126 der Verordnung vom 19. Juni 2020 (BGBl. I S. 1328) Geändert Worden ist (German Soil Protection Ordinance); Bundesgesetzblatt (Federal Law Gazette) Jahrgang 1999 Teil I, Nr. 36. https://www.gesetze-im-internet.de/bbodschv/.

[10] Piccinno F., Gottschalk F., Seeger S., Nowack B. (2012). Industrial production quantities and uses of ten engineered nanomaterials in Europe and the world. J. Nanopart. Res..

[11] Foss Hansen S., Heggelund L.R., Revilla Besora P., Mackevica A., Boldrin A., Baun A. (2016). Nanoproducts–what is actually available to European consumers?. Environ. Sci. Nano.

[12] Landi S., Carneiro J., Soares O.S.G.P., Pereira M.F.R., Gomes A.C., Ribeiro A., Fonseca A.M., Parpot P., Neves I.C. (2019). Photocatalytic performance of N-doped TiO_2_nano-SiO_2_-HY nanocomposites immobilized over cotton fabrics. J. Mater. Res. Technol..

[13] Noireaux J., López-Sanz S., Vidmar J., Correia M., Devoille L., Fisicaro P., Loeschner K. (2021). Titanium dioxide nanoparticles in food: Comparison of detection by triple-quadrupole and high-resolution ICP-MS in single-particle mode. J. Nanopart. Res..

[14] (1998). BioAbfV, Bioabfallverordnung in der Fassung der Bekanntmachung vom 4. April 2013 (BGBl. I S. 658), die Zuletzt durch Artikel 3 Absatz 2 der Verordnung vom 27. September 2017 (BGBl. I S. 3465) Geändert Worden ist. Bundesgesetzblatt (Federal Law Gazette) Jahrgang 1998 Teil I, S. 2955. https://www.gesetze-im-internet.de/bioabfv/BJNR295500998.html.

[15] Praetorius A., Tufenkji N., Goss K.-U., Scheringer M., von der Kammer F., Elimelech M. (2014). The road to nowhere: Equilibrium partition coefficients for nanoparticles. Environ. Sci. Nano.

[16] Börner R., Meiller M., Oischinger J., Daschner R. (2016). Untersuchung Möglicher Umweltauswirkungen bei der Entsorgung nanomaterialhaltiger Abfälle in Abfallbehandlungsanlagen.

[17] Oischinger J., Meiller M., Daschner R., Hornung A., Warnecke R. (2019). Fate of nano titanium dioxide during combustion of engineered nanomaterial-containing waste in a municipal solid waste incineration plant. Waste Manag. Res..

[18] Schäfer E., Holm O.E., Thomé-Kozmiensky E., Quicker P., Kopp-Assenmacher S. (2018). Beispiel einer zukunftsorientierten kommunalen Abwasserreinigung. Verwertung von Klärschlamm.

[19] (2001). Ländergemscinaft Abfall, LAGA PN98 Richtlinie für das Vorgehen bei Physikalischen, Chemischen und Biologischen Untersuchungen im Zusammenhang mit der Verwertung/Beseitigung von Abfällen. Grundregeln für die Entnahme von Proben aus Festen und Stichfesten Abfällen Sowie Abgelagerten Materialien; Ländergemeinschaft Abfall. https://www.laga-online.de/documents/m32_laga_pn98_1503993280.pdf.

[20] (1994). Länderarbeitsgemeinschaft Abfall, LAGA-Mitteilung 19, Merkblatt für die Entsorgung von Abfällen aus Verbrennungsanlagen für Siedlungsabfälle.

[21] Chen G., Liu X., Su C. (2012). Distinct effects of humic acid on transport and retention of TiO_2_ rutile nanoparticles in saturated sand columns. Environ. Sci. Technol..

[22] Wang Y., Gao B., Morales V.L., Tian Y., Wu L., Gao J., Bai W.Y.L. (2012). Transport of titanium dioxide nanoparticles in saturated porous media under various solution chemistry conditions. J. Nanopart. Res..

[23] (2009). Leaching of Solid Materials-Percolation Method for the Joint Examination of the Leaching Behaviour of Inorganic and Organic Substances.

[24] Montano M.D., Olesik J.W., Barber A.G., Challis K., Ranville J.F. (2016). Single Particle ICP-MS: Advances toward routine analysis of nanomaterials. Anal. Bioanal. Chem..

[25] Meermann B., Nischwitz V. (2018). ICP-MS for the analysis at the nanoscale–A tutorial review. J. Anal. At. Spectrom..

[26] Oischinger J., Meiller M., Daschner R., Hennecke D., Hund-Rinke K., Meisterjahn B., Schröder N. (2020). Untersuchungen zur Möglichen Freisetzung von Nanopartikeln bei der Ablagerung und Bodenbezogenen Anwendung von Mineralischen Abfällen-Abschlussbericht.

